# Arbeitnehmer:innen als Klima-Akteure: Was können Gewerkschaften gegen den Klimawandel unternehmen? Einleitung

**DOI:** 10.1177/10242589261467970

**Published:** 2026-07-21

**Authors:** Gregor Murray, Mathieu Dupuis, Julie Hagan, Mélanie Laroche, John Peters

**Affiliations:** 1 École de relations industrielles und Interuniversity Research Centre on Globalization and Work (CRIMT), Université de Montréal, Kanada; 2 Département des relations industrielles und Interuniversity Research Centre on Globalization and Work (CRIMT), Université Laval, Kanada; 3 Interuniversity Research Centre on Globalization and Work (CRIMT), Université de Montréal, Kanada; 4 Faculty of Business Administration, Department of Sociology, Memorial University

**Keywords:** Klimawandel, Gewerkschaftsstrategie, ökologischer Wandel, strategische Kapazität, gerechte Transition

## Abstract

Der Klimawandel ist eine existenzielle Bedrohung und erfordert Eindämmungs- und Anpassungsmaßnahmen, die voraussichtlich Folgen für Arbeitnehmer:innen, ihre Arbeitsplätze und ihre Gemeinden haben werden. Die Einleitung zu dieser Themenausgabe von *Transfer: European Review of Labour and Research* geht der Frage nach, wie Arbeitnehmer:innen und ihre Gewerkschaften zu Klima-Akteuren werden. Zusätzlich zu den sieben Studien in der vorliegenden Ausgabe gehen wir ebenfalls auf bereichsübergreifende Themen ein, die sich daraus ergeben. Dazu gehören die Bedeutung von gewerkschaftlicher Organisierung und Kollektivverhandlungen als Indikator für die Einstellung zu Maßnahmen gegen den Klimawandel; die strategische Vielfalt und die Forderung, auf Typologien der Klimastrategien der Gewerkschaften zu verzichten und stattdessen zu verstehen, wie diese Strategien entstehen und mit bestimmten Konstellationen von Bedingungen zusammenhängen, die durch unterschiedliche Belastungen durch Klimafolgen, divergierende Entwicklungspfade und einen asymmetrischen Zugang zu institutionellen Ressourcen bestimmt werden; die unübersichtliche Komplexität der Strategien gegen den Klimawandel; die Frage, ob gewerkschaftliche Strategien auf einer Wachstums- oder Degrowth/Postgrowth-Ökonomie, auf Erwerbsarbeit im Arbeitsmarkt oder allen Formen von Arbeit einschließlich sozialer Reproduktionsarbeit basieren; der Einfluss des Geschlechts auf Klimaverwundbarkeit und Klimaschutzaktionen; und die Versprechungen und Gefahren einer gerechten Transition.

## Einleitung

Unser Klima verändert sich. Die Menschen in der Arbeitswelt erleben unmittelbar die damit einhergehenden Auswirkungen am Arbeitsplatz und in ihren Gemeinwesen: Extremwetterereignisse (Hitzewellen, Waldbrände, Überflutungen); industrielle Verschmutzung und Bodendegradation; technologische Veränderungen und Umweltpolitik haben Auswirkungen auf ihr Leben. Für Gewerkschaften als Vertreter der Arbeitnehmer:innen ist die Erkenntnis wichtig, dass klimapolitische Maßnahmen zu Umstrukturierungen, Arbeitsplatzverlusten und geänderten Arbeitsbedingungen führen können. Gleichwohl werden Arbeitnehmer:innen und die Gewerkschaftsvertretungen zu oft von Diskussionen über Klimapolitik ausgeschlossen.

Dies ist allein deshalb ein Paradoxon, weil Arbeitnehmer:innen genau diejenigen sind, die unmittelbar mit den Folgen des ökologischen Wandels konfrontiert werden. Um die globalen Ziele für die Reduzierung der CO_2_-Emissionen zu erreichen, werden umfangreiche Investitionen und neue Produktionsprozesse erforderlich sein, die Arbeitsorganisation wird sich ändern müssen, neue Kompetenzen werden gefragt sein und staatliches Handeln muss darauf abzielen, Emissionen zu verringern und zu regulieren und das Wachstum nachhaltiger Industrien und Gemeinwesen zu fördern.

Die vorliegende Themenausgabe von *Transfer: European Review of Labour and Research* befasst sich in erster Linie mit Arbeitnehmer:innen und Gewerkschaften als Klima-Akteuren. Wir versuchen zu verstehen, wie die Gewerkschaften im Namen ihrer Mitglieder strategisch mit Umwelt- und Klimathemen umgehen. Da die Vielzahl der Strategien gut dokumentiert ist (Dupuy und Pasquier, 2024; Kalt, 2022; Thomas und Doerflinger, 2020), wird hier untersucht, welche Erklärungen es für diese Vielfalt gibt und wie Arbeitnehmer:innen und Gewerkschaften mehr Mitspracherechte erlangen können.

Wer die Rolle der Gewerkschaften in der Klimathematik verstehen will, muss beurteilen können, wie gut sie politische Möglichkeiten für sich nutzen können, um politische Entscheidungen mitzubestimmen und Arbeitgeberaktionen zu beeinflussen (Doerflinger und Thomas, 2026; Price et al., 2025; Snell und Fairbrother, 2010). Es ist ebenfalls wichtig zu betrachten, wie gewerkschaftliche Aktionen Kollektivverhandlungen neu definieren und bei den Mitgliedern zu einem besseren Verständnis für den Zusammenhang zwischen Klimaschutz- und Beschäftigungspolitik führen (Dupuis et al., 2026b; Michaud und Laroche, 2024; Schmidt und Braga, 2025). Die Priorisierung von Themen wie Klimawandel und gerechte Transition können Gewerkschaften befähigen, experimentell vorzugehen und sich neue Wirkungsmacht zu erschließen (Laroche und Murray, 2024). Im größeren Kontext eines zunehmenden Widerstandes gegen die Klimaschutzpolitik ist es wichtig zu verstehen, wie es Gewerkschaften gelingen kann, die Klima- und Industriepolitik zu beeinflussen, Entscheidungsprozesse mitzubestimmen und Visionen für Gesellschaften der Zukunft zu entwerfen.

Diese Ausgabe von *Transfer* folgt dem Auftrag einer öffentlich engagierten Forschung und will unser Verständnis der gewerkschaftlichen Strategien für die Eindämmung des Klimawandels und zur Anpassung an dessen Folgen auf Ebene der Arbeitsplätze, Branchen und Wirtschaftssektoren vertiefen. Auf der Grundlage konzeptioneller Innovationen, von Fallstudien und vergleichender Forschung möchten wir das Wissen für eine profundere Diskussion darüber vermitteln, wie arbeitnehmergeführte Organisationen den Klimawandel bekämpfen können und dabei gleichzeitig Qualitätsarbeitsplätze und gerechtere Gemeinschaften entstehen.

Das übergeordnete Ziel besteht darin, Denkprozesse darüber anzuregen, wie Arbeitnehmer:innen zu Klima-Akteuren werden können und welche Rolle die Gewerkschaften in diesem Prozess spielen können. Die Forschung braucht hierzu neue Wissensinitiativen, die auf den Realitäten der jeweiligen Branchen und Betriebe basieren. Für die Gewerkschaften ist ein intensiverer Wissensaustausch und eine weitere Auseinandersetzung mit der Klimathematik erforderlich, um sich für eine Industrie-, Kollektivverhandlungs- und Fortbildungspolitik einzusetzen, die den Arbeitnehmer:innen und ihren Gemeinschaften zugutekommt.

## Einblick in die Strategien von Arbeitnehmer:innen und Gewerkschaften gegen den Klimawandel

Das Team der Gastredakteure hat eine Reihe von Autor:innen, die über Arbeitnehmer:innen, Gewerkschaften und den Klimawandel forschen, gebeten ihre Beiträge vor dem Hintergrund der Erfahrungen mit unterschiedlichen Formen von Arbeit, Branchen und nationalen Kontexten vorzulegen und daraus Erkenntnisse zu gewinnen, wie sich Arbeitnehmer:innen und ihre Gewerkschaften mit der Klimathematik auseinandersetzen. Die nachfolgenden sieben Beiträge untersuchen sowohl konzeptuell als auch empirisch und komparativ, wie Arbeitnehmer:innen und ihre Gewerkschaften mit den Klimaherausforderungen umgehen.

Der Klimawandel hat nicht nur profunde Herausforderungen in Form von Arbeitsplatzverlusten, Neuorganisation von Arbeit und Gesundheitsrisiken zur Folge, sondern bietet auch potenzielle Chancen für neue Arbeitsplätze und Branchen. In diesem Kontext sind die Beschäftigten und ihre Gewerkschaften zu oft nur passive Adressaten staatlicher oder unternehmerischer Entscheidungen. In ihrem Versuch, Arbeitnehmer:innen und Gewerkschaften in ihrer Eigenschaft als Klima-Akteure zu verstehen, schlagen Mathieu Dupuis, Julie Hagan, Mélanie Laroche, Gregor Murray und John Peters (2026b) einen Rahmen vor, um die Dynamik des klimarelevanten Handelns (oder dessen Fehlen) von Gewerkschaften in der Klimapolitik zu verstehen.

Der Artikel untersucht zunächst einige der Strategien, die die Gewerkschaften verfolgen könnten. Anhand des Grades der Zustimmung zur Klimapolitik oder ihrer Ablehnung und der Stärke des konkreten Engagements für den Klimaschutz haben sie fünf allgemeine Strategien benannt. Diese lassen sich bezeichnen als sich enthaltend, bedingungslos unterstützend, bedingt unterstützend, oppositionell und transformativ. Nachdem sie diese abhängige Variable identifiziert hatten (*was unternehmen die Gewerkschaften gegen den Klimawandel*), haben die Autor:innen ein exploratives Rahmenkonzept vorgeschlagen, um die Faktoren zu verstehen, die diese Strategien potenziell beeinflussen. Dieses Rahmenkonzept verbindet interne Faktoren wie Arbeitnehmerinteressen, gewerkschaftliche Machtressourcen und strategische Fähigkeiten der Gewerkschaften mit externen Einflüssen wie struktureller Macht, institutioneller Macht, Diskurs innerhalb der Gesellschaft und Arbeitgeberstrategien. Damit verfolgen die Autor:innen zwei Ziele: für Forschende, plausible Zusammenhänge zur Erklärung bestimmter Gewerkschaftsstrategien zu untersuchen; für Gewerkschaften und andere Akteure, potenzielle Hebel zu verstehen, um in der Klimapolitik mehr bewirken zu können.

Ein wichtiges Thema in den Debatten über Arbeitnehmer:innen und ihre Einstellung zu Fragen des Klimaschutzes betrifft die Frage, inwieweit die Klimastrategien der Gewerkschaften nach wie vor dem Archetyp des männlichen Industriearbeiters folgen. Dieser Fragestellung gehen Jane Parker, Valeria Pulignano und Bianca Luna Fabris (2026) nach. Sie untersuchen das Spannungsfeld zwischen ökologischer Nachhaltigkeit, Geschlechtergleichstellung und weiter gefassten wirtschaftlichen Entwicklungsverläufen. Die Klimaschutzstrategien der Gewerkschaften basieren in der Regel auf einer durch Arbeit und Technologie bestimmten Produktivität als Mittel, um Wirtschaftswachstum zu generieren, die Früchte des Wirtschaftswachstums umzuverteilen und Vollbeschäftigung sowie menschenwürdige Arbeit zu erreichen. Anders gesagt, setzen die Klimaschutzstrategien der Gewerkschaften meistens auf Wirtschaftswachstum und Produktivismus. Was aber, wenn die Klimakrise – wie oftmals zu hören ist (Räthzel, 2025) – eher Degrowth- und Postgrowth-Strategien erfordert und nicht immer weiteres Wachstum? Ein solcher Paradigmenwechsel würde nicht nur bisher vorherrschende ökonomische Lehrmeinungen in Frage stellen, sondern auch die geschlechterstereotypen Annahmen, die ein fester Bestandteil gewerkschaftlicher Strategien sind. Dadurch würde sich eine Tür zu alternativen Vorstellungen von Erwerbsarbeit, Care-Arbeit und Nachhaltigkeit öffnen.

Für Parker et al. (2026) müssen sich gewerkschaftliche Ansätze zum Klimawandel diese kritischere Perspektive zu eigen machen, indem sich die Gewerkschaftsstrategien gegenüber der feministischen politischen Ökonomie und dem Ökofeminismus öffnen. In der Erkenntnis, dass der Produktivismus einen steigenden Energie- und Materialeinsatz zur Folge hat, propagieren Parker et al. eine neue Sicht auf das Verhältnis zwischen Produktions- und sozialer Reproduktionsarbeit und berücksichtigen dabei die weiteren Auswirkungen auf das Familien- und Gemeinschaftsleben sowie die geschlechterspezifische Arbeitsteilung. Diese neue Deutung unseres Verständnisses von Gewerkschaftsarbeit und Klimawandel nach diesen Kriterien beinhaltet zwei miteinander verknüpfte konzeptuelle neue Sichtweisen, um gendergerechte gewerkschaftliche Ansätze zum Klimawandel zu entwickeln. Erstens muss der Blick über Produktivismus und Wirtschaftswachstum hibausgehen; und zweitens müssen soziale Reproduktionsarbeit und bezahlte Erwerbstätigkeit als Gesamtkonzept gesehen werden, wobei beide Elemente für eine gerechte Transition gleichermaßen bedeutend sind. Solch ein Verständnis einer gerechten Transition erfordert ein Bewusstsein für Vielfalt und als wichtigste Ziele das Wohlergehen der Beschäftigten und soziale Gerechtigkeit, wobei die Institutionen der Arbeitsbeziehungen wirtschaftliche Anforderungen, ökologische Imperative und Diversität miteinander verbinden.

Vor dem Hintergrund einer erstarkenden populistischen und extrem rechten Politik und eines orchestrierten politischen Widerstandes gegen die Klimapolitik wird das Ausmaß der öffentlichen Unterstützung für den Umweltschutz ein wichtiger Faktor für das Verständnis der gewerkschaftlichen Strategien. Wenn Gewerkschaftsmitglieder und potenzielle Mitglieder die von ihren Gewerkschaften befürwortete Politik zur Bewältigung des Klimawandels nicht mittragen, dann ist diese Opposition eine kritische Determinante für die Wirksamkeit der Gewerkschaften beim Kampf gegen den Klimawandel. Diese Überlegungen haben Josef Ringqvist (2026) veranlasst, das Verhältnis zwischen den Institutionen der Arbeitsbeziehungen und der Unterstützung der Öffentlichkeit für den Umweltschutz zu analysieren. Durch einen Abgleich von Daten des European Social Survey mit dem neuen Europäischen Partizipationsindex (EPI) untersucht Ringqvist, ob länderübergreifende Unterschiede in der Stärke von Gewerkschaften und Institutionen der Arbeitsbeziehungen länderspezifischen Unterschieden der öffentlichen Zustimmung zur CO_2_-Besteuerung entsprechen. Diese Unterstützung wird als Indikator für die Einstellung zum Klimawandel gewertet. Auf der Basis vorhergehender Arbeiten über den positiven Zusammenhang zwischen der Geltungsreichweite von Kollektivverhandlungen und der umweltschutzaffinen Einstellung von Arbeitnehmer:innen lässt seine statistische Mehrebenenanalyse einen ähnlichen positiven Zusammenhang zwischen den Institutionen der Arbeitsbeziehungen und der öffentlichen Unterstützung für eine CO_2_-Steuer erkennen.

Eine der aussagestärksten Branchen, in der sich die Initiativen der Gewerkschaften für die Ausgestaltung der Dekarbonisierung nachvollziehen lassen, ist die Automobilindustrie mit ihrem Übergang zur Elektromobilität. Diese gewerkschaftlich stark organisierte Branche bietet die Möglichkeit für fundierte analytische Vergleiche. Die Studie von Ian Greer und Grzegorz Lechowski (2026) vergleicht die Verhandlungsrunde der United Auto Workers 2023 und der Detroit Three als größte US-Automobilhersteller mit den Verhandlungen zwischen der IG Metall und Volkswagen in Deutschland im Jahre 2024. Die Autoren argumentieren, dass hier entscheidend ist, die Aktionen und Strategien der Gewerkschaften in den spezifischen politisch-ökonomi-schen Kontext der Umstellung der Produktionssysteme in der Branche, der unternehmerischen Strategien und der wettbewerblichen Zwänge zu stellen. Auf der Basis von Interviews mit den zwei wichtigsten Gewerkschaften im Automobilsektor in diesen beiden Ländern gehen sie der Frage nach, wie diese Gewerkschaften verschiedene Strategien verfolgt und unterschiedliche Machtressourcen genutzt haben, um die Umstellung auf Elektromobilität in ihrem Sinn zu gestalten. Dieser Vergleich macht deutlich, wie nationale Institutionen, Entwicklungspfade in der Autoindustrie und gewerkschaftliche Kapazitäten insgesamt die Möglichkeiten für die Arbeitnehmerschaft bestimmen, die industrielle Dekarbonisierung zu gestalten.

Beide Gewerkschaften haben zu Streiks aufgerufen, jedoch mit deutlich unterschiedlichen Ergebnissen: Wo die UAW sich robust durchgesetzt hat, musste die IG Metall beträchtliche Konzessionen akzeptieren. Ungeachtet der bestehenden Unterschiede der Institutionen und der politischen Ökonomie wird die Umstellung auf die Elektromobilität durch Konflikte über sich ändernde Unternehmensstrategien und politische Maßnahmen der Regierung gekennzeichnet. Greer und Lechowski (2026) weisen auf eine Infragestellung der kooperativen Systeme der Arbeitsbeziehungen in der deutschen Automobilindustrie sowie auf eine zunehmend konfliktorientierte Gewerkschaftsstrategie in den USA hin. Sie haben festgestellt, dass die größere organisatorische und institutionelle Macht der IG Metall nicht zu einem besseren Ergebnis geführt hat. Die höhere Konfrontationsbereitschaft der UAW war allerdings auch nicht der einzige Grund für ihren Erfolg. Die Verhandlungsergebnisse wurden durch externe Faktoren wie zum Beispiel die ausgeprägte hochrangige politische Unterstützung der Elektromobilität, volatile Produktmärkte infolge des Wettbewerbsdrucks durch ausländische Importe, ausländische Direktinvestitionen (ADI) und die Gefahren von Verlagerungen an kostengünstigere Produktionsstandorte bestimmt.

In ihrer Studie über Möglichkeiten der Gewerkschaften für strategische Antworten auf die Dekarbonisierung der Industrie untersuchen Benjamin Crawford, Marion Dumas, Fergus Green, Xaquín Garcia und Katja Treichel-Grass (2026) die Maßnahmen zur Transformation der Automobilindustrie in Deutschland, Spanien und dem Vereinigten Königreich. Die Antworten der Gewerkschaften auf ein langfristiges „Verbrenner-Aus“ müssen gleichzeitig auch Klima- und Umweltschutz sowie industriepolitische Themen mitdenken und Einfluss auf die Entscheidungen multinationaler Konzerne und zahlreicher Unternehmen in den Lieferketten berücksichtigen, die an der Produktion beteiligt sind. Der Klimawandel stellt Gewerkschaften vor Herausforderungen, die weit über die traditionellen Themenbereiche von Kollektivverhandlungen und des sozialen Dialogs hinausgehen. Für Crawford et al. (2026) ist der Kapazitätsaufbau innerhalb der Gewerkschaften der wichtigste Aspekt dieser Herausforderung. Ungeachtet institutioneller Unterschiede sehen sie einen gemeinsamen Nenner strategischer Kapazitäten mit entscheidender Bedeutung für eine effektive Antwort der Gewerkschaften auf den Klimawandel. Dazu gehören die Fähigkeit, vorausschauend zu handeln, einen Ausgleich zwischen divergierenden Interessen zu finden, glaubwürdige Strategien zu formulieren und effektiv in größeren politischen und grundsatzpolitischen Szenarien zu agieren. Wenn Gewerkschaften diese Fähigkeiten haben, könnten sie die Erfolgsaussichten für die Dekarbonisierung und für einen gerechten Übergang verbessern. Eine fundierte Politikgestaltung, so argumentieren sie, sollte die Entwicklung dieser Fähigkeiten und der dazu erforderlichen Wissensregimes bei den Gewerkschaften unterstützen.

Welche Einstellungen Arbeitnehmer:innen zum Klimawandel haben, kann nicht von den umfassenderen politisch-ökonomischen Verhältnissen getrennt werden, unter denen sie leben und arbeiten. Dies ist die wichtige Erkenntnis einer Vergleichsstudie der gerechten Übergänge in Deutschland und Südafrika, vorgelegt von Vera Trappmann, Ruth Bookbinder, Alex Beresford und Felix Schulz (2026). Auf Basis von Interviews mit Gewerkschafter:innen, Umweltaktivist:innen und politischen Entscheider:innen mit unmittelbarer Beteiligung an Initiativen für eine gerechte Transition in den beiden Ländern vergleichen sie die Erfahrungen und Perspektiven von Arbeitnehmer:innen und Bevölkerungsgruppen, die am stärksten von der Umstellung auf eine klimabewusste Politik und die Dekarbonisierung der Industrie betroffen sind. Während es in Deutschland eine breite Unterstützung für eine Politik zur Bewältigung des Klimawandels gibt und sich Arbeitnehmer:innen und ihre Gewerkschaften aktiv und Seite an Seite mit der Regierung und Klimaaktivist:innen für eine Klimaschutzpolitik engagieren, ist in Südafrika die Idee einer „gerechten Transition“ zu einer Quelle von Zynismus, Misstrauen und Ängsten im Energiesektor geworden. Das geht so weit, dass Gewerkschaften aktiv mit Kampagnen versuchen, diese Transition zu stoppen oder zu verhindern. Trappmann et al. (2026) stellen dieser ohne eigene Kontrolle durchgeführten „gerechten Transition“ im Globalen Süden die selbst gesetzte Agenda des Globalen Nordens entgegen. In Südafrika kann, mangels einer überzeugenden Alternative durch internationale Solidarität zwischen Gewerkschaften und Aktivist:innen im globalen Süden und Norden, eine gerechte Transition nicht von der fragwürdigen und spaltenden rassistischen Politik der postkolonialen Zeit getrennt betrachtet werden. Daran wird deutlich, dass Maßnahmen zur Bewältigung des Klimawandels nie einfach nur technische oder wirtschaftliche Prozesse sind, sondern zutiefst politische Auseinandersetzungen, geprägt durch eine Historie der Ungleichheit, institutionelle Vermächtnisse und miteinander konkurrierende Vorstellungen von Gerechtigkeit.

Viviana Asara, Marco Caligari und Emanuele Leonardi (2026) betrachten ebenfalls den Fall von Arbeitnehmer:innen und ihren Gewerkschaften in fossilabhängigen Energie- und Industriesektoren, hier anhand eines Kohlekraftwerkes in Civitavecchia, Italien. Dass der Markt bei der Umsetzung des ökologischen Wandels versagt, so argumentieren sie, zeigt umso deutlicher, wie wichtig die Mitsprache der Arbeitnehmer:innen, der Gewerkschaften und der Bevölkerung bei der Ausarbeitung von Strategien für Klimagerechtigkeit ist. Ein wichtiges Thema müssen deshalb die Bedingungen sein, die Handeln für den Klimaschutz als sinnvollere Maßnahme erscheinen lassen als Widerstand gegen den Klimaschutz. Im Wesentlichen geht es um die Frage, wie Arbeitnehmer:innen und ihre Gewerkschaften ihre oft defensiven Positionen, erklärbar aus den strukturellen Zwängen perpetuierter fossiler Strukturen (Carbon Lock-In), überwinden und sich mit einer schrittweisen Umstellung auf grüne Energien anfreunden könnten.

In dem analysierten Beispiel aus Italien hat ein heterogenes Bündnis von Gewerkschaften und Umweltgruppen eine umfassende Zusammenarbeit und Koordinierung erreicht und in der Folge ihre Strategie des defensiven Widerstandes aufgegeben und fordert jetzt eine gerechte Transition, bei der bessere Arbeit und eine saubere Energieinfrastruktur im Mittelpunkt stehen. Auf der Grundlage qualitativer Daten und der Analyse von Dokumenten haben Asara et al. (2026) die Dynamik einer auf Koalitionen beruhenden Macht analysiert, die für die Erneuerung der Gewerkschaften und für die Beseitigung der strukturellen Privilegien des in fossilen Energieträgern investierten Kapitals entscheidend war. Dieser Fall ist aufgrund der Bottom-Up-Teilnahme so interessant, weil die Gewerkschaften hier eine führende Rolle in den Sozialverhandlungen auf der lokalen Ebene übernommen haben und sich offen für eine gerechte Transition stark gemacht haben. Sie argumentieren, dass demokratische Initiativen der Gewerkschaften auf lokaler Ebene erfolgreich politische Untätigkeit auf nationaler Ebene überwinden und die Umsetzung politischer Maßnahmen zur Entstehung hochwertiger Arbeitsplätze und ökologischer Nachhaltigkeit vor Ort forcieren können. Das Autorenteam weist auf die Bedeutung zweier Faktoren hin: erstens auf die strukturellen Auswirkungen der Transition auf Arbeitnehmer:innen und ihre Gemeinschaften; und zweitens auf die Notwendigkeit einer doppelten Konvergenz bei der Bildung von Bündnissen. Dies ist intern zwischen Gewerkschaftsfunktionär:innen, der Gewerkschaftsbasis und unterschiedlichen Arten von Gewerkschaften erforderlich, und extern mit Umweltbewegungen und der Zivilgesellschaft insgesamt. Allerdings gibt es keine einfachen Lösungen, da auch Wissens- und Kompetenzerweiterung und institutionelle Unterstützung benötigt werden, damit Gewerkschaften eine transformative Rolle spielen können.

## Welche Erkenntnisse können wir aus diesen Beiträgen gewinnen?

An erster Stelle steht sicherlich, dass *Arbeitnehmer:innen und ihre Gewerkschaften unverzichtbare Akteure des ökologischen Wandels sind*. Die Artikel in dieser Ausgabe von *Transfer* zeigen deutlich, wie die Auswirkungen des Klimawandels die Arbeitnehmer:innen und ihre Gewerkschaften in das Zentrum dieser Transformation rücken. Traditionelles Unternehmensmanagement schließt die Belegschaften allzu oft von wichtigen Entscheidungen über Investitionen, die Übernahme neuer Technologien, die Neuorganisation der Produktion, die Qualifizierung der Beschäftigten und die Nutzung ihres Knowhows zu diesem Zweck aus. Obwohl die Anpassung an den Klimawandel und die Verringerung der CO_2_-Emissionen die Mitwirkung der Arbeitnehmer:innen und der Gewerkschaften erfordern, werden diese Gruppen oft von Klimaschutz-Entscheidungsprozessen ausgeschlossen. In diesem Kontext versuchen die Gewerkschaften, Arbeitnehmerinteressen zu schützen und sich für sie einzusetzen. Darüber hinaus scheint der gewerkschaftliche Organisationsgrad einen wichtigen Einfluss auf die Einstellung der Arbeitnehmer:innen gegenüber dem Klimawandel zu haben – dies wird in öffentlichen politischen Debatten und in der Frage, wie Staaten häufig Maßnahmen zur Dekarbonisierung angehen, allzu oft vernachlässigt. Ringqvist (2026) weist auf eine eindeutige Korrelation zwischen der Mitgliedschaft in einer Gewerkschaft und der öffentlichen Unterstützung für eine CO_2_-Steuer hin. Dies deutet auf eine komplexe Beziehung zwischen Institutionen der Arbeitsbeziehungen, dem Vertrauen in gesellschaftliche Institutionen und der Beteiligung der Zivilgesellschaft hin (Bolet et al., 2024; Turner et al., 2020). In einer Zeit, in der die europäische Politik sich erneut der Auswirkungen eines abnehmenden gewerkschaftlichen Organisationsgrades und einer geringeren Geltungsreichweite von Kollektivverhandlungen auf gesellschaftliche Ungleichheiten bewusst wird, scheint die Mitgliedschaft in einer Gewerkschaft einen wichtigen Einfluss darauf zu haben, inwieweit Arbeitnehmer den ökologischen Wandel unterstützen. Es handelt sich hier um eine komplexe Beziehung, aber die Bedeutung der Stimme der Belegschaften und der Gewerkschaften für den ökologischen Wandel ist eine wichtige Erkenntnis.

Zweitens *ist die Mitwirkung der Arbeitnehmer:innen und ihrer Gewerkschaften an der Entwicklung von Maßnahmen zur Bewältigung der Klimakrise sehr unterschiedlich und uneinheitlich*. Während einige Gewerkschaften die zur Bekämpfung des Klimawandels vorgeschlagenen Lösungen ablehnen – sei es im Namen des Arbeitsplatzschutzes oder weil es ein aussichtsloses Unterfangen sei, den Kapitalismus vor sich selbst zu retten – argumentieren andere, dass es dringend notwendig sei, sich zu engagieren, denn „auf einem toten Planeten gibt es keine Arbeitsplätze“. Diese Gewerkschaften konzentrieren sich auf die Arbeitsbedingungen und die Gesundheit der Arbeitnehmer:innen sowie auf die Reduzierung von Treibhausgasen durch die Einführung umweltfreundlicherer Technologien und die Vermittlung neuer Qualifikationen. Mit anderen Worten: Einige Gewerkschaften lehnen klimapolitische Maßnahmen ab; andere versuchen, den Prozess zu verzögern, um ihre Partikularinteressen zu verteidigen; wieder andere entscheiden sich für eine transformative Strategie. Auch wenn die Vielfalt der verschiedenen Gewerkschaftsstrategien mittlerweile typologisch gut ausdifferenziert ist, befindet sie sich noch in einem vorläufigen „Benennungsstadium“. Insgesamt wissen wir jedoch weniger darüber, welche Faktoren zu der einen oder anderen Strategie beitragen und welche Faktoren einen bestimmten strategischen Ansatz beeinflussen könnten. Dies ist ein wichtiger Beitrag von Dupuis et al. (2026b), die versuchen, eine Reihe von Akteuren zu identifizieren, die bestimmte Strategien fördern oder hemmen. Dies ist auch der primäre Fokus der detaillierten empirischen Studien in verschiedenen nationalen Kontexten, die in dieser Ausgabe veröffentlicht werden (Asara et al., 2026; Crawford et al., 2026; Greer und Lechowski, 2026; Trappmann et al., 2026).

Drittens, *Gewerkschaften müssen in der Lage sein, ihre Macht zu mobilisieren*. Um eine bekannte Erkenntnis der Gewerkschaften aufzugreifen: „Wenn Arbeitnehmer:innen nicht mit am Tisch sitzen, stehen sie auf der Speisekarte.“ Arbeitnehmer:innen und ihre Gewerkschaften können nur als Klima-Akteure tätig werden, wenn sie ihre Machtressourcen entwickeln und ausspielen können. Es gibt durchaus eine Reihe von Faktoren, die sie in die Lage versetzen oder daran hindern, diese Ressourcen zu mobilisieren. Die in dieser Ausgabe veröffentlichten Artikel beschreiben unterschiedliche Machtressourcen, die entweder intern bei den Gewerkschaften vorhanden sind oder extern in Anspruch genommen werden können. Von besonderem Interesse sind die strategischen Kapazitäten von Gewerkschaften, da sie diese selbst fördern und entwickeln können (Lévesque und Murray, 2010). Einige Beiträge bieten wichtige Erkenntnisse über diese Kapazitäten und ihre Rolle bei der Förderung eines größeren gewerkschaftlichen Engagements (Asara et al., 2026; Crawford et al., 2026; Dupuis et al., 2026b). Sind diese Fähigkeiten dagegen nicht vorhanden, kann dies zu Strategien der Enthaltung oder der bedingungslosen Unterstützung führen. Crawford et al. (2026) sehen ebenfalls eine Rolle für die Politik bei der Entwicklung von gewerkschaftlichen Wissensregimes im Bereich des Klimawandels. Informierte, gut ausgestattete und strategisch geschickte Gewerkschaften sind in einer besseren Position, Wege zur Dekarbonisierung zu gestalten und dafür zu sorgen, dass die Arbeitnehmerinteressen während dieses Wandels umfassend berücksichtigt werden.

Viertens *wird bei der Auseinandersetzung mit der unübersichtlichen Komplexität des Klimawandels in unterschiedlichen Betrieben und Branchen deutlich, wie vielfältig die einzelnen Fälle wirklich sind.* Dies ist als Warnung vor einer zu schnellen Verallgemeinerung zu verstehen, da zum Beispiel institutionalistische Erklärungsansätze unsere Sichtweise auf Gewerkschaftsstrategien zu stark festlegen. Greer und Lechowski (2026) weisen darauf hin, wie wichtig es ist, Gewerkschaftsstrategien in den Kontext spezifischer politischer Ökonomien und ihrer eingebetteten Produktionssysteme, Unternehmensstrategien und Wettbewerbszwänge zu setzen. In ihrem Vergleich zwischen Südafrika und Deutschland betrachten Trappmann et al. (2026) die asymmetrische Integration nationaler politischer Ökonomien in die Weltwirtschaft und die unterordnende Wirkung, die dieses Machtungleichgewicht auf Gewerkschaftsstrategien für die Energieerzeugung unter Verwendung fossiler Brennstoffe in Südafrika hat. Es muss nicht nur die Bedeutung einer Reihe von Faktoren beurteilt werden (darunter natürlich Institutionen, Technologien, Märkte, das Risiko des Arbeitsplatzverlustes, die Macht der Gewerkschaften sowie strategische Kapazitäten), sondern das Zusammenwirken dieser Faktoren erfordert eine detaillierte Analyse, um es zu verstehen. Arbeitsplätze und Branchen sind höchst unterschiedlich und können sich in der Tat kontraintuitiv auf Strategien zur Bekämpfung des Klimawandels auswirken (Dupuis et al., 2026a).

Fünftens *wird der Entwicklungspfad zu einer kohlenstoffarmen Gesellschaft voraussichtlich durch erhebliche Konflikte markiert werden*. Die vorherrschenden Machtstrukturen insbesondere im Zusammenhang mit fossilen Energieträgern sowie die zunehmende Bedeutung klimafeindlicher populistischer Bewegungen sind nicht förderlich für Initiativen gegen die Klimakrise. Darüber hinaus tragen sie zur Verstärkung von Ungleichheiten und zur Schwächung demokratischer Institutionen bei. Gewerkschaften haben das Potenzial, sich diesen Herausforderungen unter der Voraussetzung zu stellen, dass sie sich flexibel in der komplexen Dynamik des externen politischen und ökonomischen Umfeldes bewegen und dabei gleichzeitig ihre eigene interne Dynamik als Organisation fördern. Dies kann zu erheblichen Konflikten mit Arbeitgebern, dem Staat oder zivilgesellschaftlichen Organisationen führen, aber voraussichtlich auch zu neuen Bündnissen mit diesen Akteuren.

Sechstens *neigen Gewerkschaften dazu, ihre Strategien nach Wachstumszielen zu konzipieren.* Degrowth- oder Postgrowth-Strategien, die auch Gerechtigkeitsaspekte berücksichtigen, bieten hier einen alternativen Politikrahmen. Parker et al. sehen dies als eine Möglichkeit an, das Verhältnis zwischen Wirtschaftswachstum und Produktivität sowie das Verhältnis von Arbeit im Erwerbsarbeitsmarkt und oftmals nicht bezahlter Care-Arbeit und sozialer Reproduktionsarbeit radikal zu überdenken.

Schließlich *ist das Konzept einer „gerechten Transition“ ein wichtiges Kapitel im Textbuch der Gewerkschaften zum Klimawandel geworden, wird jedoch in der Gewerkschaftsbewegung unterschiedlich interpretiert*. Der Ursprung dieses Konzepts liegt in der ökologischen Kehrtwende der US-Gewerkschaften in den 1970er Jahren, die zu wichtigen Fortschritten im Arbeits- und Umweltschutz geführt haben. Gewerkschaften haben dieses Konzept seitdem in wichtige Dokumente zum Klimawandel aufgenommen. Die Internationale Arbeitsorganisation (IAO) hat es in ihren Leitlinien für ökologische Nachhaltigkeit und menschenwürdige Arbeit berücksichtigt, und es findet sich auch in der Präambel des Pariser Klimaabkommens von 2025. Die Arbeitnehmer:innen müssen eine unverhältnismäßig hohe Belastung durch die Transitionskosten schultern – in Form von Arbeitsplatzverlusten, aufgezwungenen Ortsveränderungen, zunehmender Vulnerabilität und der Notwendigkeit von Unterstützung während der Bewältigung des Klimawandels. Im Lichte der Studien in dieser Themenausgabe und der Tatsache, dass der Begriff „gerechte Transition“ zu einem Streitpunkt geworden ist, stellt sich die Frage, ob dieses Etikett heute eher als Oxymoron zu bezeichnen ist und nicht als Sammelruf für Arbeitnehmerorganisationen. Eine gerechte Transition steht für die Ökologisierung der Wirtschaft „in einer Weise, die so gerecht und inklusiv wie möglich für alle Betroffenen ist, Möglichkeiten für menschenwürdige Arbeit bietet und niemanden zurücklässt“ (ILO, 2018). Gerechtigkeit und Inklusion sind wichtig für eine gerechte Transition, da Arbeitnehmer:innen multiplen Risiken ausgesetzt sind. Es gibt ebenfalls die langfristige Aufgabe, grundlegende Rechte bei der Arbeit wie zum Beispiel die Versammlungsfreiheit, ordnungsgemäße Verfahren und gleichen Schutz aller durch das Gesetz zu verteidigen (Stevis, 2023).

Um die Prioritäten einer gerechten Transition umzusetzen – starke Einflussmöglichkeiten für Arbeitnehmer:innen und ihre Gewerkschaften, ihre Beteiligung an Entscheidungen, adäquate öffentliche Politik und Unterstützung sowie Fairness –, müssen sich Arbeitnehmer:innen und ihre Gewerkschaften einer Vielzahl signifikanter Herausforderungen stellen und ihre Kapazitäten stärken. Wir hoffen, dass diese Themenausgabe einen Beitrag zum Erreichen dieses Ziels leistet.
